# Rapid response to meningococcal disease cluster in Foya district, Lofa County, Liberia January to February 2018

**DOI:** 10.11604/pamj.supp.2019.33.2.17095

**Published:** 2019-05-29

**Authors:** Julius Monday Rude, Lavele Kortimai, Fallah Mosoka, Baller April, Mouhamoud Nuha, Victoria Katawera, Thomas Nagbe, Alpha Tamba, Williams Desmound, Richard Mulbah, Formenty Pierre, Emmanuel Musa Onuche, Joseph Okeibunor Chukwudi, Ambrose Talisuna, Ali Ahmed Yahaya, Soatiana Rajatonirina, Tolbert Nyenswah, Bernice Dahn, Alex Gasasira, Ibrahima Socé Fall

**Affiliations:** 1 World Health Organization, Monrovia, Liberia; 2 Ministry of Health, Voinjama, Liberia; 3 National Public Health Institute, Monrovia, Liberia; 4 US Centers for Disease Control and Prevention, Monrovia, Liberia; 5 World Health Organization, Geneva, Switzerland; 6 World Health Organization, Regional Office for Africa, Brazzaville, Congo; 7 Ministry of Health, Monrovia, Liberia

**Keywords:** Meningococcal disease, Klemabendu, Foya, Lofa, meningitis belt, rapid response

## Abstract

**Introduction:**

Early detection of disease outbreaks is paramount to averting associated morbidity and mortality. In January 2018, nine cases including four deaths associated with meningococcal disease were reported in three communities of Foya district, Lofa County, Liberia. Due to the porous borders between Lofa County and communities in neighboring Sierra Leone and Guinea, the possibility of epidemic spread of meningococcal disease could not be underestimated.

**Methods:**

The county incidence management system (IMS) was activated that coordinated the response activities. Daily meetings were conducted to review response activities progress and challenges. The district rapid response team (DRRT) was the frontline responders. The case based investigation form; case line list and contacts list were used for data collection. A data base was established and analysed daily for action. Tablets Ciprofloxacin were given for chemoprophylaxis.

**Results:**

Sixty-seven percent (67%) of the cases were males and also 67% of the affected age range was 3 to 14 years and attending primary school. The attack rate was 7/1,000 population and case fatality rate was 44.4 % with majority of the deaths occurring within 24-48 hours of symptoms onset. Three of the cases tested positive for Neisseria Meningitidis sero-type W while six cases were Epi-linked. None of the cases had recent meningococcal vaccination and no health-worker infections were registered.

**Conclusion:**

This cluster of cases of meningococcal disease during the meningitis season in a country that is not traditionally part of the meningitis belt emphasized the need for strengthening surveillance, preparedness and response capacity to meningitis.

## Introduction

Meningococcal meningitis has been observed worldwide with the highest number of cases occurring in the meningitis belt of sub-Saharan Africa, that stretch from Senegal in the west to Ethiopia in the east (26 countries). For over 100 years, major epidemics of meningococcal disease have occurred every few years within the African meningitis belt with the most recent large-scale epidemic occurring in 2012 with over 22 000 cases and 1931 deaths [1]. The global burden of meningococcal disease has not been well documented due to inadequate surveillance system and documentation in some parts of the world. *Neisseria meningitides* serogroup A (Nm A) has been the cause of the majority of invasive meningococcal infections in the meningitis belt, although other strains such as serogroups C, X and W have also caused epidemics [1], and other pathogens such as *Haemophilus influenzae* type b (Hib) and *Streptococcus pneumoniae* (Spn) are also responsible for bacterial meningitis cases [1, 2]. Large meningococcal disease outbreaks caused by serogroup W have occurred in Burkina Faso in 2002 , recurred 10 years later; Burkina Faso in 2012, Niger (2010-2011), Chad (2009-10) and Guinea (2013). In all these outbreaks the proportion of infections among younger children (less than 10 years) was high [1, 3]. Liberia, a non-meningitis belt country shares border with Guinea and Ivory Coast which are in the meningitis belt. Meningococcal carriage, which represents the first step of disease transmission, varies with age and setting. It is known that *Neisseria meningitides* colonizes the nasopharynx in up to 5-10% of adults who are asymptomatic [4]. A recent study demonstrated that the carriage prevalence increases throughout childhood from 4.5% in infants to a peak of 23.7% in 19 year old subjects, then decreases to 7.8% in 50 year old adults [5]. The first time Liberia experienced a meningitis outbreak which was associated with attending a funeral in Sinoe County was in April 2017 where fourteen individuals with unknown illness including eight deaths were reported and *Neisseria meningitides* sero type C pathogen was the identified cause [6, 7]. *Neisseria meningitides* is transmitted from person-to-person through droplets of respiratory or throat secretions from carriers. Smoking, close and prolonged contact-such as kissing, sneezing or coughing on someone, mass gatherings or living in close quarters with a carrier-facilitates the spread of the disease .The bacteria can be carried in the throat and sometimes overwhelms the body´s defenses allowing the bacteria to spread through the bloodstream to the brain. It is believed that 1% to 10% of the population carries *Neisseria meningitides* in their throat at any given time. However, the carriage rate may be higher (10% to 25%) in epidemic situations [1, 3]. The average incubation period is two to four days, but can range between two and 10 days [3, 4, 8]. The most common symptoms are stiff neck, high fever, and sensitivity to light, confusion, headaches and vomiting. In addition in infants, excessive crying, bulging fontanels and limp body are commonly found. A less common but even more severe (often fatal) form of meningococcal disease is meningococcal septicemia, which is characterized by a hemorrhagic rash and rapid circulatory collapse [9, 10]. If untreated, meningococcal meningitis is fatal in 50% of cases and may result in brain damage, hearing loss or disability in 10% to 20% of survivors [4]. On January 13, 2018, Foya district health team (FDHT) notified the Lofa County Health Team (LCHT), the National Public Health Institute of Liberia (NPHIL) and the World Health Organization (WHO) of a cluster of unexplained health events that involved 9 cases including 4 deaths from three communities in Lofa County of Northern Liberia. These communities are located in a northern triangle consisting of Liberia bordering Sierra Leone and Guinea. On January 15, 2018, a team of National Public Health Institute of Liberia (NPHIL) and World Health Organization (WHO) epidemiologists were deployed from national level to support the county-led field investigation and response. Epidemiologic investigations performed included active case finding, determining the magnitude of the outbreak, and ascertaining the cause of unknown illness at that time. On January 22, 2018, *Neisseria meningitides* sero type W was confirmed by RT-PCR at National Reference Laboratory of Liberia (NRL) in specimens (oral swab, cardiac fluid and whole blood) collected from 3 cases. Cerebrospinal fluid (CSF) was not collected and tested due to lack of specimen collection materials and inadequate skills to perform lumbar puncture by health workers in the peripheral health facilities where the case patients were managed from. Ebola virus, Lassa fever, Yellow fever, Typhoid fever, Hepatitis A & C pathogens were ruled out. Response measures that included surveillance and active case search, contacts listing and follow up, appropriate clinical management of cases, chemoprophylaxis with ciprofloxacin, infection prevention and control, social mobilization and engagement with traditional healers, leaders of places of worship, local leaders; and dead body management were rapidly implemented to control the outbreak and prevent new cases and deaths. Meningitis surveillance in Liberia is implemented through the integrated disease surveillance and response system (IDSR), which captures priority diseases and conditions including unexplained clusters of health events and deaths [11, 12] reported from health facilities to the district, then up to the county and to the national level. This paper aims at describing the rapid response activities that were conducted to contain a cluster of meningococcal disease event in a remote part of the country within close proximity to both Ivory Coast and Guinea which are Meningitis belt countries.

## Methods

**Figure 1:**
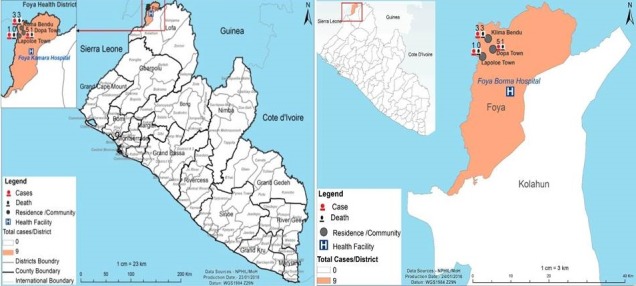
Map of Liberia showing Lofa County on left and on the right Map of Foya district affected areas

**Figure 2:**
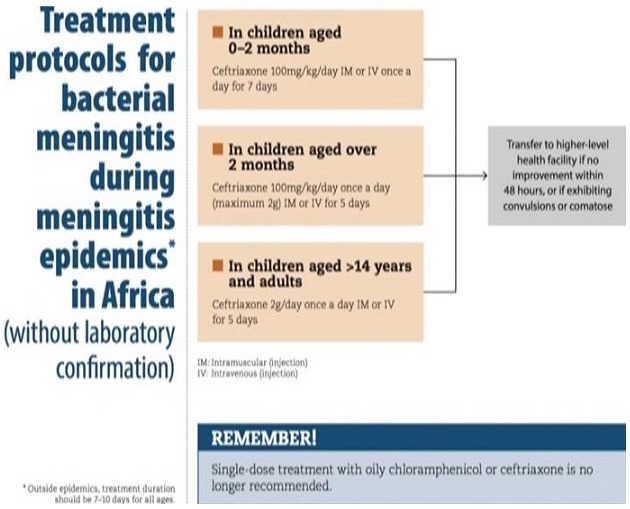
Bacterial meningitis protocol

**Setting:** Lofa County is one of 15 counties in Liberia with a total population of 358,613, located in northern region of the country, approximately 110 miles from the capital Monrovia and neighbouring countries of Guinea and Sierra Leone. Its many porous borders [13, 14] allowing free population movements, poor access to health services and inadequate safe water supply may increase the risk of outbreaks. In the last five years, the county has had outbreaks ranging from measles, Lassa fever, yellow fever, Ebola, acute bloody diarrhea (ABD) and cholera [13, 15].The inhabitants are predominantly farmers. The county has 59 health facilities (04 hospitals, 04 health centres and 51 primary clinics) with Telleweyan Memorial Hospital as the county referral hospital located in Voinjama city. Kelimabendu town the Epi- community is located at the triangle of the borders between Liberia, Guinea and Sierra Leone, and has an estimated population of 517 inhabitants. It is one of the catchment communities of the Mendikoma Clinic in Foya District, Lofa County. At Mendikoma Clinic, about 50% of the patients that seek care come from neighboring Sierra Leone and Guinea (Figure 1, Figure 2).

**Case definitions:** case definitions used included: 1) the initial case definition for **unexplained cluster of deaths:** two or more people in the same community who die suddenly of unknown or infectious cause after suffering similar symptoms as investigations were under way to establish the causes. 2) **Health facility case definition:** any person coming from or visiting Lofa County and presenting with two or more of the following symptoms: headache, vomiting, general body weakness, confusion, fever and among children, persistent crying, refusal to eat, fixed gaze, rigid body from December 23, 2017. 3) **Community case definition:** any person coming from or visiting Lofa County who is not feeling well from December 23, 2017.

**Data collection:** we used the data collection tools for outbreak response in Liberia, including the IDSR case based investigation form, line-list form, contact listing and daily follow-up form which were used to collect epidemiological, clinical, and demographic information on cases and their contacts [11]. District surveillance officers, district environmental health officers, district health officer, county surveillance officer conducted the case investigations and active case finding, with field supervision from WHO and national-level epidemiologists. The team interviewed patients and their relatives, community members, contacts, local leaders, traditional leaders and health facilities staff to understand circumstances and characteristics of the event.

**Figure 3:**
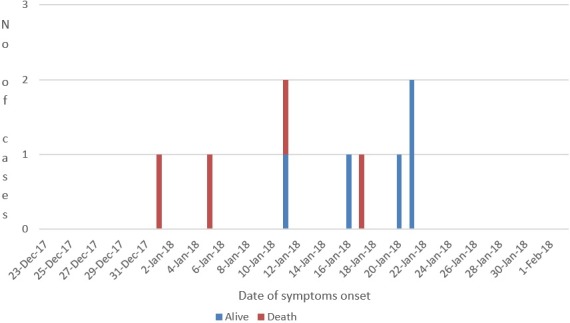
Epicurve of menigococcal disease outreak, Foya District, Lofa County, 2017/2018

**Record review:** data on clinical symptoms consistent with the case definition were reviewed for individuals seeking care during three months (October - December 2017) from outpatient department (OPD) registers, the IDSR ledger and inpatient case management charts and registers at all health facilities in Foya district. This was done to detect any cases that could have been unreported (Figure 3).

**Case management:** cases were managed at Foya Borma Hospital isolation unit. WHO managing meningitis epidemics in Africa; used for case management for this outbreak in Lofa County, Liberia [16].

**Data analysis:** all data were analyzed using Epi Info^TM^ 7.0. Data from the IDSR case based investigation form, line-list form, patient medical records, contact listing and daily follow-up form which were used to collect epidemiological, clinical, and demographic information on cases and their contacts was analysed and the cluster event described for appropriate public health actions.

## Results

**Figure 4:**
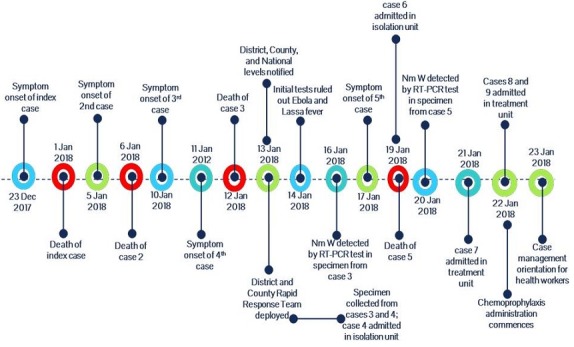
Timeline of events of meningococcal disease outbreak, Foya District, Lofa County, Liberia, 23 December 2017 – 29 January 2018

Figure 4 shows the total cluster event cases since Jan 1, 2018 were 9 including 4 deaths (CFR: 44.4%). The index case died on 1st January 2018 however the cluster event was reported from the district to county and national level after death of the 3rd case that occurred 11th January 2018. The frequency of new cases based on the date of symptom onset peaked on January 21, 2018 with 3 new cases admitted on that day. 90% of the deaths occurred within 24-48 hrs of symptoms onset. Most cases and deaths were clustered around Kelimabendu the Epi-index community for the event. 90% of the cases were close family members from 03 households and attended the same place of worship. Most of the cases were students 06 (67%) from the same school which posed serious risk of spread among the pupils. There were no travel outside Liberia among cases and no significant associations observed with other potential exposures analyzed. The turn-around time for meningococcal disease laboratory results was on average 24 to 48 hours following the deployment of a vehicle for transportation of specimens from the field (Lofa County) to the national reference laboratory (NRL) in Margibi county, however the initial inability to suspect meningococcal disease among the index and second cases had an effect of delay in confirmation of the disease, initiation of appropriate treatment protocol and public health control measures. Nine (09) cases were line listed in one month period of which 6 (67%) were males. The median age affected was 12, range 3-85 years and 70% of the cases were aged 3-14years. Six (67%) cases were school going age children while 1(11%) was a pre-school child (Table 1).

**Table 1:** Demographic data and geographical distribution of cases

Patient demographics			Occupation	Geographical distribution of affected communities in Foya district, Lofa county			Cases classification	
Initials of case	Sex (M:F)	Age (years)	Occupation	Kelimabendu-	Dopa	Laypalloe	Confirmed	Epi-Link
J N	M	45	Farmer and water pump mechanic	1(Died)				1
JJN	M	3	Not yet in school	1(Died)				1
TN	F	6	Primary school-student	1(Died)			1	
TJN	M	13	Primary school-student	1				1
J N	M	12	Primary school-student		1(Died)		1	
SK	M	85	Stays home (elderly)		1			
SC	F	12	Primary school-student		1			
MYN	F	8	Primary school-student			1	1	
JN	M	4	Kindergarten pupil		1			
Total	6M			4	4	1	3	6
	3F			9			9	

**Figure 5:**
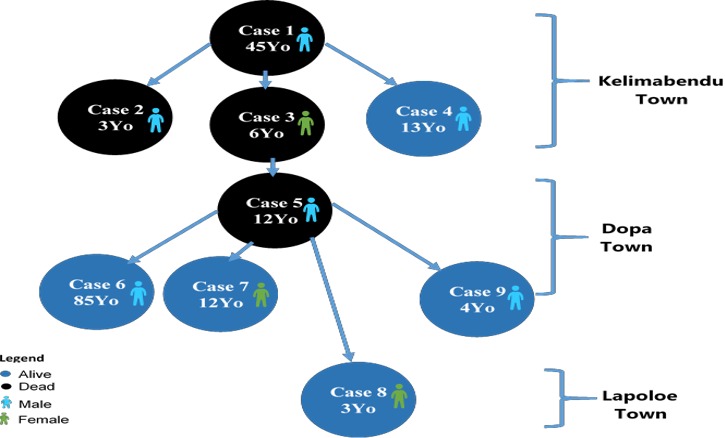
Transmission chain

A total of 237 contacts ware followed daily for 10 days. As a result of active contact follow up, 4 cases were identified and early treatment initiated resulted in good prognosis. No health worker contacts developed symptoms (Table 2). Single dose oral ciprofloxacin 500 mg chemoprophylaxis was given to 233/237(99%) contacts, 103 non-contact health workers and 843 none contacts residents of affected communities; none of the contacts that took chemoprophylaxis developed symptoms of the meningococcal disease none of the contacts was pregnant or, a lactating mother. The attack rate was 7/1,000 population and the case fatality rate was 44.4% in January 2018 and the risk of getting meningococcal disease during January 2018 among the three ringed communities was very high in Dopa the last community to register cases (Table 3). Transmission involved 3 families in 3 closely linked communities (Figure 5).

**Table 2:** Meningitis contact tracing, Foya District, Lofa County, 2018

Variables	TOTAL	Kelimabendu	Dopa	Laypalloe	Ndendu	Foya Town	Kolahun Town
Total contacts listed	237	82	72	45	6	25	7
Number (%) of contacts seen daily for 10days	237(100%)	82(100%)	72(100%)	45(100%)	6(100%)	25(100%)	7(100%)
% of contacts lost to follow up	0%	0%	0%	0%	0%	0%	0%
Not seen [number]	0	0	0	0	0	0	0
Total Health Care Worker Contacts	32	1	1	0	0	23	7
Number of cases identified through contacts follow up	4	0	3	1	0	0	0
Contacts completed 21 days (Graduated)	237(100%)	82(100%)	72(100%)	45(100%)	6(100%)	25(100%)	7(100%)
Number of Health care workers who became ill	0	0	0	0	0	0	0

**Table 3:** Meningococcal disease attack rate per 1,000 populations in affected communities

Affected community	Community population	Number of cases	Attack rate per 1,000 population
Kelimabendu	517	4	8
Dopa	85	4	47
Laypalloe	689	1	1
**Total**	1291	9	7

One patient developed increased intracranial pressure, a complication of meningitis and was managed with Mannitol (0.25g/kg IV bolus over 5 min), furosemide (1 mg/kg IV bolus), dexamethasone to 10 mg IV qid, 30 % head elevation, urine output monitoring and nil by mouth (NBM), intravenous (i.v) maintenance fluids of 0.9% saline and 5% glucose to correct any potential hypoglycemia on addition to ceftriaxone. Oxygen was not available for supportive care and Lumbar puncture which is contraindicated in such cases was avoided. The index case 45 years old symptoms on set was on 23rd December 2017, however the case patient only sought care on 1st January 2018 and died the same day and subsequently three of his children became symptomatic and only one survived the illness. The last cases symptoms onset was 22nd January 2018; they were treated at Foya Borma hospital isolation unit and recovered. The meningococcal disease Lofa cluster ended after recording no new suspected or confirmed case and all line listed contacts finishing their ten days follow up.

**Public health response:** the Lofa County health team coordinated the investigation and response to the event with support from WHO and NPHIL. The County incidence management system (IMS) and Foya district RRT were activated and met daily to review field reports and progress of implementation of response activities. At the national level, the National Public Health Institute of Liberia (NPHIL) is providing technical and operational support to the county with support from WHO and US Center for Disease Control and Prevention. A total of 30 general community health volunteers (gCHVs) with supervision of district health officer, county surveillance officer and WHO epidemiologist conducted house to house active case search in the affected communities and ring health facilities to identify additional cases on addition to following up contacts two times a day for 10 days. A database was created to manage the epidemiological, clinical and laboratory data (Line list, contacts list and Laboratory results). Cross border engagement activities included 5 POE visited to as part of surveillance heightening process and information sharing with local health officials in neighboring communities in Sierra Leone and Guinea was regularly done. Infection prevention and control (IPC) measures were put in place (distribution of hand washing buckets, teaching sessions on respiratory hygiene and cough etiquette, avoiding close contact with sick people, and community engagement) as a means of breaking the chain of transmission. IPC standards and protocols were emphasized in affected communities, health facilities, public places and at points of entry. Use of risk appropriate personal protective equipment (PPE) by healthcare workers was reinforced, assessments for PPE availability in all health facilities in Foya district were conducted and all health facilities had adequate IPC supplies. Critically-ill patients were admitted to Foya Borma hospital isolation unit to reduce the likelihood of disease transmission. Persons with suspected or confirmed *Neisseria meningitides* infection were hospitalized at Foya Borma hospital isolation Unit. Immediate treatment with appropriate antibiotics (intravenous Ceftriaxone) according to case management protocol was initiated as after collection of blood specimens and this approach improved prognosis. One of the cases lost her sight as complication of the disease. Technical, medical supplies and logistics support were provided by WHO & NPHIL/MOH.

In a bid to ensure adequate compliance to case management protocol and patient care, a total of 63 healthcare workers from Foya district were trained in the use of case management protocol for meningococcal disease while 38 health workers working at Foya Borma hospital (doctors, physician assistants and nurses) were oriented in management of complications of meningococcal disease. A total of 1,179 people (233 contacts, 103 non-contact health workers and 843 non contacts residents of affected communities) received single ciprofloxacin tablets as chemoprophylaxis as one of the quickest available prevention measures at the time. Meningococcal reactional vaccination was not carried out because the vaccine was not available in the country and the cluster size was considered small. Despite this idea; ministry of health remains focused to increase vaccination coverage against meningococcal disease in the country as the main prevention measure. Community engagement with local leaders, traditional healers, clan leaders and leaders of 17 places of worship (02 mosques and 17 churches) were critical in social mobilization and awareness to the public on addition to gCHV and community health development committee members that conducted house to house awareness. Information was also provided to the public through radio talk shows and street broadcasters, encouraging ill persons in the community to seek care at health facilities and encouraging community members on IPC practices. Two thousand five hundred (2,500) community members were sensitized on simplified case definition (syndromic) for meningococcal disease The index case and the second case were buried by the community while the subsequent cases that passed on were accorded safe and dignified burial by a trained district burial team supervised by the county environmental health officer. The community members who conducted the initial burials were placed on the contacts list received chemoprophylaxis and was under observation for 10 days. None of them developed signs and symptoms of the disease. The affected families and communities ware offered psychosocial support and counseling by a team of mental health clinicians in dealing with the emotional stress of the sudden death of their relatives and social stigma that developed. Health workers were also supported to emotionally respond to the event.

## Discussion

The rapid response to the cluster of illnesses and deaths in Lofa County is a reflection of the increased public health and outbreak response capacity established in Liberia during and after Ebola epidemic and highlights the importance of enhanced surveillance systems [17], increased diagnostic capacity of the public health laboratory system and specimen referral; designated and trained rapid response teams, enhanced communications and information systems for outbreak response and the existence of a public health emergency operations center (EOC) as effective measures to prevent widespread disease outbreaks and other public health events [6, 18, 19]. In 2014, an initial cluster of illnesses and deaths resulting from EVD took more than 90 days from detection to coordination of the emergency response and led to a widespread Ebola epidemic [20]. In contrast, response efforts for this cluster of illnesses and deaths were initiated in less than 48 hours of detection by Lofa county health team, NPHIL, WHO and CDC. However, the CFR was very high (44%). Although the CFR is usually high in sub-Saharan Africa, reaching sometimes more than 60% [21], early diagnosis and treatment can reduce dramatically the number of deaths during meningococcal disease outbreaks [5, 22] to reach the level of the developed countries [23]. A study conducted in Niger in 2015 suggested that the CFR was 14.8% due to use of antimicrobial prophylaxis for the contacts like in Lofa County after the detection of the outbreak [24]. Meningococcal diseases typically include meningococcal meningitis and to some extent meningococcal bloodstream infection (septicemia). Patients with acute meningococcemia may present with meningitis, meningitis with meningococcemia, or meningococcemia without clinically apparent meningitis [25, 26]. A person can present with one or both features, as was the case with the cluster event in Lofa County unlike what was seen in Sinoe County southeast Liberia in May 2017 which was typically the septicemic type [6]. Clinicians worldwide need to be on the lookout for both types of manifestations in suspected meningitis cases. Meningococcal disease epidemics like any other outbreak are very disruptive, requiring the establishment of emergency treatment centers and placing a severe strain on the routine health services. The reason for the susceptibility of this region of Africa to major epidemics of meningococcal disease is in part related to its climatic features, with outbreaks occurring mainly in the hot, dry season [2]. During the dry season between December to June, dust winds, cold nights and upper respiratory tract infections combine to damage the nasopharyngeal mucosa, increasing the risk of meningococcal disease. At the same time, transmission of *Neisseria meningitides* may be facilitated by overcrowded housing [1, 2].

The fact that 67% of affected people were school age children posed a risk of spread among the school children due to overcrowding in the classroom and close contacts during playing at school. This event was similar to the case of Northern Nigeria and Niger in 2017 where the disease also mostly affected children between the ages of 5-14 years; making it more deadly since these age groups have lower immunity [27]. During these challenging periods, Liberia’s Laboratory system demonstrated its preparedness to provide timely feedback for some basic tests post Ebola outbreak. The laboratory quickly ruled out Ebola, yellow fever and Lassa fever and confirmed *Neisseria meningitides* serotype “W” by RT-PCR. This helped to initiate appropriate effective case management and supportive treatment that increased survival among patients. For this cluster, cerebrospinal fluid (CSF) was not tested due to lack of specimen collection materials and inadequate skills to perform lumbar puncture by health workers in the peripheral health facilities. Lumbar puncture is recommended to be performed in all suspected cases with clinical signs and symptoms of invasive meningococcal disease (IMD) except in patients with prolonged seizures, immunocompromised patients, in the presence of signs of space-occupying lesions and in patients with severe impairment of consciousness and shock. In most cases; cerebrospinal fluid (CSF) reveals high opening pressure, pleocytosis, high protein levels and low glucose levels [28]. *Neisseria meningitides* should be detected in the CSF or blood by Gram staining, standard culture and/or polymerase chain reaction (PCR) [27, 29, 30]. Some of the best practices noted during the response activities and appreciated during the post action review meeting include; timely situation report sharing with national level, partners, neighboring counties and countries (Sierra Leone & Guinea), administration of Chemoprophylaxis to contacts and non-contacts, massive awareness on local radio stations, market places and places, involvement of traditional, local and places of worship leaders in community engagement and social mobilization and distribution of simplified messages on signs & symptoms including preventive measures to mosques and churches as well as the timely provision of essential drugs by national public health institute of Liberia and WHO. During this cluster, it was noted that cases were not initially identified and reported early by community based surveillance due to lack of community health assistance in affected communities while at the primary health facilities cases were misdiagnosed due to inadequate knowledge on case definition of meningococcal disease, isolation facility was not fully prepared for the isolation and management of cases, limited infection prevention and control implementation at community level and inaquate laboratory capacity for basic general investigations like complete blood cell count and skills to collect appropriate specimens (e.g. Lumbar puncture for CSF) in rural health facilities affected the response.

Lessons learned: 1) prompt community engagement before, during, and after the outbreak and provision of regular feedback created high level of trust between the community and the response team; 2) recruitment of CHVs from their own community to conduct contact tracing enhance their cooperation and improve good surveillance practice; 3) administration of antibiotic prophylaxis and best IPC practices helped to prevent transmission of the disease; 4) the multi-sectorial collaboration resulted in the implementation of appropriate steps to prevent further transmission, and WHO determined the risk of recurrence of the meningococcal disease outbreak as low.

**Recommendations:** 1) the districts and county review and validate the epidemic preparedness and response plan (EPR) at the end of every outbreak; 2) the National Public Health Institute of Liberia (NPHIL) and ministry of health preposition IDSR sample collection materials including CSF collection kits to all hospitals in Liberia; 3) the County, MoH and partners re-activate the community based surveillance in areas where community health assistants (CHAs) are not assigned; 4) cross border surveillance activities should be prioritized by boarder counties and regular local information sharing among districts of neighboring countries encouraged; 5) clinicians worldwide need to be on look for both pictures in suspected meningitis cases, the IDSR and meningitis clinical management literature may require updating to future this possibility; 6) as part of preparedness the need to prioritize meningococcal vaccination to populations at high risk and health workers is key despite its cost implication; 7) the occurrence of a second health event of meningococcal disease in anon meningitis belt country during the meningitis outbreak season suggests the inclusion of Liberia in the belt.

## Conclusion

The successful response to this cluster demonstrates the increased capacity of Liberia’s public health system to rapidly detect and effectively respond to public health threats and enhance global health security. The need to prioritize stocks of meningitis vaccines, laboratory supplies and ensuring epidemiologic surveillance and response systems are to public health events is in place through CRRTs and DRRTs in Liberia are established, are key health capacities Liberia is contributing to global health security. Implications of this documentation: clinicians worldwide need to be on lookout for both features in suspected meningitis cases, the IDSR and meningitis clinical management literature may require updating on this possibility while the occurrence of a second health event of Meningococcal disease in a non- meningitis belt country during the meningitis outbreak season suggests the inclusion of Liberia in the belt. As part of preparedness the need to prioritize meningococcal vaccination to populations at high risk and health workers is key despite its cost implication.

### What is known about this topic

- Infection with Neisseria Meningitidis easily progress to Meningococcal Sepsis, also known as Meningococcemia, causing a rash, hemorrhage, and multi-organ failure as was the case in the Sinoe county-Liberia situation;
- Neisseria Meningitidis serotype C was identified.

### What this study adds

- Documentary evidence for the infection progression with both Meningitidis and Meningococcemia features occurring together as was the case in this event;
- Neisseria Meningitidis serotype W was identified by RT-PCR for the first time in Liberia.

## Competing interests

The authors declare no competing interest.
